# Single strain versus multi-strain probiotics in aquaculture: comparative impacts on gut microbiota and immune responses in fish and shellfish

**DOI:** 10.3389/fimmu.2026.1895127

**Published:** 2026-08-13

**Authors:** Nandini Rai, Ankit Kachore, J. M. Julka, Bimal P. Mohanty, Smruti R. Sahoo, Fan-Hua Nan, Sofia P. Das

**Affiliations:** 1 Shoolini University, Solan, India; 2 Siksha O Anusandhan, Bhubaneswar, India; 3 Department of Aquaculture, National Taiwan Ocean University, Keelung, Taiwan

**Keywords:** aquaculture immunity, fish immune response, gut microbiota, multi-strain probiotics, probiotics, single-strain probiotics

## Abstract

Probiotics have gained wide popularity as environmentally sustainable replacement for antibiotics in aquaculture due to their ability to up-regulate the host immune competence and boost disease resistance. However, considerable knowledge is yet to gain regarding differential efficacies and mechanisms of actions of Single-strain vs multi-strain probiotics. In the present review, the differences between the immunomodulatory potentials of Single-strain and multi-strain probiotics in fish and shellfish were thoroughly compared, with focus on immune gene expression of immunity markers and alteration of gut microflora. Single-strain probiotics usually mount a targeted immune response by enhancing the expression of particular immunity parameters, whereas multi-strain probiotics generally interact synergistic to induce more general and enhanced immune response and greater microbial diversity in the gut. Molecular basis and mechanisms underpinning probiotic mediated immunomodulation were also comprehensively documented in this review. Although recent evidence demonstrate that multi-strain probiotics tend to offer a broader and consistent immune defence against pathogens than Single-strain formulations, the efficacy of probiotic supplements is often context-specific, depending on the rearing environmental condition, dosage, strains compatibility etc. The combination of probiotics and biofloc system has been proposed as a promising and eco-friendly technology for the development of the sustainable aquaculture and animal welfare. Finally, existing research gaps and future directions have also been discussed for sustainable aquaculture.

## 1 Introduction

Demand for animal protein has soared, and as such, aquaculture has quickly become one of the fastest growing food production sectors worldwide contributing significantly to economic growth and food security. Farmed fish and seafood now account for more than half of the fish consumed globally, an indispensable addition to our modern food supply (1). However, the boom in production has come at a cost; the shift towards intensive farming, characterized by high stocking densities, feed reliance on artificial sources, and ecological stress, have increased the susceptibility of aquatic species to pathogenic infections caused by bacteria, viruses and parasites (2). Diseases not only affect profitability but also pose a genuine threat to the sustainability and efficiency of aquaculture as a whole industry (3).

Antibiotics and chemotherapeutic drugs have been the prevailing methods to control and prevent diseases in aquaculture over the years (4), yet continued use has now raised significant issues. The continuous and widespread use and abuse of antibiotics has rapidly accelerated the evolution and spread of antimicrobial resistance (AMR), a serious global health issue (5), residual chemical substances in fish pose a risk to human health, while accumulation in water systems perturbs microbial ecology and reduces species richness (6). Therefore, the need to identify and explore safe and sustainable alternatives has been widely recognized by the aquaculture industry (7).

Probiotics are increasingly recognized as a viable alternative to traditional antibiotics in aquaculture. They are defined as live microorganisms that provide health benefits to the host when provided in adequate amounts (8) and have been found to enhance growth performance, feed efficiency and disease resistance in various fish species and crustaceans. The favourable effects are attributed mainly to the modulation of the gut microbial communities, the prevention of pathogen colonization, the synthesis of antimicrobial substances and the induction of immune host responses (9–11). The digestive tract of fish and shellfish consists of highly complex communities of microbes which are important in the defense against disease (12). The gut microbiota plays an important role in metabolism, digestion of food and immune signaling, the effective regulation of which, by shifting these microbial communities in favor of beneficial bacteria by excluding the harmful ones (13), is crucial in strengthening the host immunity against disease (14). Despite various merits, there are many drawbacks for probiotic organisms. Viability of probiotics in storage, during feed processing, their consistency in the commercial farming and compatibility between strains, can differ depending on their origin, the species treated and external environment, dose, and culture condition. Introducing the probiotic into the fish farming ecosystem may affect the endemic microorganisms. Henceforth, more research needs to develop the ideal formulations for best use of probiotics.

**Figure 1:**
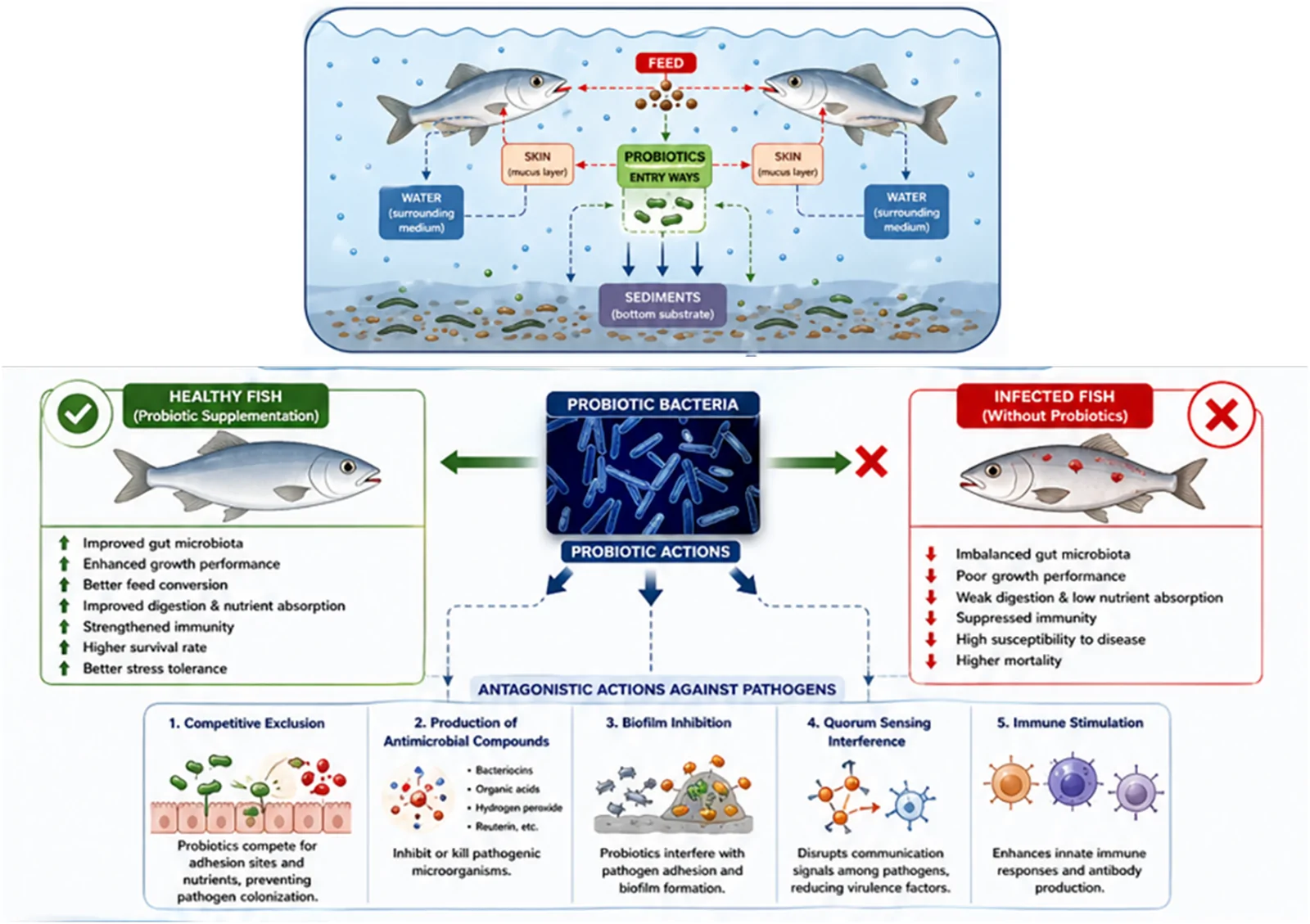
Summarizes the major routes of probiotic administration and their mechanisms of action, highlighting their role in improving gut health, immune responses, growth performance, and disease resistance in aquaculture species.

There are two categories of probiotics, namely, single-strain probiotics (SSPs) and multi-strain probiotics (MSPs) (**Figure 1**) commonly used in aquaculture. SSPs, defined by one microbial strain exhibiting a specific function, produce predictable results in controlled conditions, for instance, *Lactobacillus plantarum, Bacillus subtilis*, and *Saccharomyces cerevisiae* (15). These SSPs generally provide positive impacts via targeting a single beneficial function, for example, generating antimicrobial metabolites, secreting enzymes and stimulating immune response (16). Conversely, MSPs are consist of more than two suitable micro-organisms that are known to have mutual activities or synergies among the members of the microbial community (17).

Combining different micro-organisms, MSPs enhance the establishment of intestinal micro flora, utilization of nutrient, elimination of pathogens, nutrient metabolism and immune response (18, 19). Despite the application and benefits have already been explored in fish farming, the effectiveness and immunomodulation mechanisms comparison have yet to fully elucidate. Although, SSP and MSP have recently been explored for use in aquaculture, their comparative ability in modulating fish and shellfish immune system has not been investigated in great detail (20). In majority of these studies either SSP or MSP alone were assessed, whereas the comparison studies assessing MSP versus SSP side by side for immune modulating activity on similar experimental setting is scarce (21).

Fish and shellfish utilize distinct immune systems that are vital in the defence against infection. The immune system of fish involves innate and adaptive immunity, while shellfish, lack a robust adaptive immunity and primarily utilize an innate immune system (22). Probiotics have long been utilized to augment immune responses through alterations of immune gene expression related genes and pathways that promote increased disease resistance (23). While both the utilization of both SSPs and MSPs have demonstrated effectiveness in improving immune function, the underlying mechanisms are poorly elucidated, as is the most effective method (24, 25). The integration of probiotics into novel aquatic culture techniques such as BFT also shows significant promise, although comparative data of SSP and MSP combined effects on host immunity is minimal (26–28).

The purpose of this review is to provide a critical and comparative analysis between SSPs and MSPs on their immunological benefits towards fish and shellfish. This review will explore the mode of action of probiotics, effect on gut microbiota, immunomodulation of genes responsible for immunity, advantages and disadvantages of using SSPs or MSPs, present research gaps and propose future avenues such as the formulation of novel consortia and its application in sustainable aquaculture.

## 2 Immune systems of fish and shellfish

The watery environments fish and shellfish inhabit are packed with both beneficial and pathogenic microorganisms. Consequently, fish and shellfish have highly evolved immune systems that provide protection and defend against disease (29). Constant exposure has also led to a very heavy reliance on the innate immune response particularly by shellfish. While fish employ both innate and adaptive immune responses shellfish are generally considered to rely almost solely on innate immunity for their primary protection (30).

### 2.1 Innate immunity

Fish and shellfish use innate immunity as a primary and highly effective means of rapidly clearing potential invaders and pathogens. While the innate immune system is non-specific it is highly effective at identifying and destroying a vast number of different microbes by employing a range of physical barriers, specialized cells and humoral components (31). Physical barriers such as the skin, scales and mucus coating in fish provide a crucial first layer of protection. Mucus, far from being simply a lubricant, contains an array of active molecules such as antimicrobials, enzymes and immunoglobulins that help neutralize and prevent pathogens from infecting the fish (32). In shellfish this role falls mainly on the exoskeleton and epithelial layer. As these are inherently more permeable then fish skin their use relies on their high internal immunity for defense against invading pathogens (33).

Beyond physical barriers, innate immunity relies on the function of specialized cells. Macrophages, considered lead phagocytes in fish, are critical in destroying bacteria, secreting cytokines that recruit other immune cells and presenting antigens, thereby bridging the gap between innate and adaptive immunity. Neutrophils, in a similar vein, migrate quickly to infected tissues where they destroy bacteria by phagocytosis and release reactive oxygen species (34, 35). Haemocytes play a analogous role in the immune response of shellfish, by engulfing invading pathogens, encapsulating larger targets and releasing anti-microbial factors (36).

The effectiveness of the innate immune system is also amplified by soluble factors (humoral components) found in body fluids. The enzyme lysozyme breaks down the cell walls of bacteria and is often used as an indicator of the fish’s general health in studies of aquaculture. Other components such as complement enhance the removal of pathogens through opsonization and stimulate inflammation, while anti-microbial peptides can defend against viruses, fungi and bacteria (37–39). All these humoral factors provide a quick response to potential threats in aquatic environments.

### 2.2 Adaptive immunity

Compared with the rapid response of the innate immune system adaptive immunity is highly specific and long-lasting due to the creation of immune memory allowing for an accelerated and enhanced response when encountering a known pathogen. However, the extent and presence of these mechanisms differ widely between fish and shellfish (40).

Fish are capable of developing an adaptive immune response that is considered far less sophisticated then higher vertebrates. This system involves B cells producing specific antibodies, namely immunoglobulin M (IgM), which neutralize and clear pathogens (41). T cells, meanwhile, perform cell-mediated immunity by orchestrating the general immune response and identifying and killing infected cells. Although IgM is the dominant antibody found in fish blood and mucus that is used to detect invasive pathogens, adaptive immunity is considerably slower and has far less long-lasting memory than is seen in mammals, making innate immunity a crucial aspect of fish immunity (42).

Shellfish on the other hand do not have a traditional adaptive immune response, using instead their innate immune systems to defend themselves (41). Haemocytes are the main cells mediating immunity and can engulf and destroy the pathogens by phagocytosis and encapsulation as well as releasing anti-microbial factors. While it has been observed that certain shellfish can develop immune priming following an earlier exposure to pathogens, thus enhancing future responses, this is thought to be separate from the adaptive immunity found in higher animals (43).

### 2.3 Immune gene markers as functional indicators

On the molecular level the immune systems of fish and shellfish are controlled by genes that relate to immunity, these can serve as useful indicators of the host’s condition and of the efficiency of probiotics or other immunostimulants in aquaculture systems (44). The expression of pro-inflammatory cytokines is critical in initiating and controlling the immune response; such cytokines include interleukin-1, tumor necrosis factor and interleukin-6 and are crucial in identifying and eliminating threats. The inflammatory response needs to be controlled by the secretion of anti-inflammatory cytokines like interleukin-10 and transforming growth factor- so as not to damage tissues (45). The recognition of pathogens by Pattern Recognition Receptors (PRR), namely Toll-Like Receptors (TLR), is crucial in the initiation of the inflammatory pathway by signaling down a cascade of adapter proteins (MyD88) and transcription factors (NF-κB) to initiate immune gene expression. By identifying and monitoring the gene expression level can determine the interaction between the host and invading microbe and understand underlying biological mechanisms (46).

Fish and shellfish possess a well-organized complex network of physical barriers, specific cells and molecular elements that provide protection from pathogens, thus constitute the immune system of these aquatic animals. This immune defense involves innate immunity, a highly and vitally important line of defense in shellfish while the animal having both innate and adaptive immunity is an advantage to fight specifically against pathogen. The complex genetic regulatory machinery has also to be addressed to mediate and to sustain this host defense (22). The manipulation of such an immune system requires the basic knowledge and precise control over various immune mechanisms for the future development and enhancement of probiotic therapies to augment resistance against diseases and animal health and sustainability in aquaculture sector.

## 3 Single- strain probiotics

Single strain probiotics are a prominent choice in aquaculture research due to their definite function, ease of administration, and predictability. In aquaculture these probiotics are employed to treat fish and shellfish based on specific biological pathways to improve host immunity and function. A common method within both fin and shellfish culture involves administration of a single probiotic species to enhance immune capability, growth rate, and host survival against pathogens (47).

Single strain probiotics are specific supplements consisting of a single, known microbial strain that contributes well defined health benefits. These probiotics are developed to thrive in the intestinal tract, adhere to the intestinal walls, and exhibit immune stimulating and antimicrobial producing functions (15). Some of the most frequently used species in aquaculture include *Bacillus* species, Lactobacillus species, and yeast such as *Saccharomyces cerevisiae*. *Bacillus* species are particularly advantageous for the host’s resistance in the intestinal tract because endospores will survive in the stressful environments associated with high salinities, fluctuations in temperature, and a lack of nutrients. Conversely, *Lactobacillus* species stimulate immunity by producing a large amount of lactic acid to reduce the colonization of pathogenic bacteria. *Saccharomyces cerevisiae* is a species that is added to fish feed for the production of certain components that contribute to immunity and increase digestibility. *Saccharomyces cerevisiae* includes cellular components such as beta glucans which contribute to host immune stimulation (48). These single strain probiotic systems are effective treatments for cultivated fish due to their relatively consistent response when given to a variety of aquatic animals.

One of the most impressive features of single strain probiotic systems are their ability to modulate expression of host immune related genes within the fish and shellfish. Studies have demonstrated that addition of the correct probiotic strain into the diet may cause up-regulation of key pro-inflammatory cytokines [IL-1β], tumor necrosis factor-alpha [TNF-α]. These cytokines serve as a primary response signaling molecule necessary for generating effective innate immune responses against infections (49). When fed to fish such as common carp (*Cyprinus carpio*) the single strain *Bacillus subtilis* elevated expression of key immune genes (IL-1β, TNF-α). There was a subsequent elevation in the numbers of immune cells and a significant improvement in the lysozyme levels as well as increased phagocytic activity. For Tilapia (*Oreochromis niloticus*), inclusion of the single strain *Lactobacillus plantarum* elicited improved immune gene expression in comparison to control feed, including a notable increase in IL-1β and TNF-α levels. These results suggest that feeding a single probiotic strain enhanced immune responses and the fish’s defences against certain bacterial pathogens (50). In shrimp, specifically *Litopenaeus vannamei*, *Bacillus* species have also enhanced immune gene expression along with phenoloxidase activity and overall survival against specific pathogenic strains. In both of these studies, the yeast probiotic *Saccharomyces cerevisiae* enhanced immune responsiveness in the host by stimulating peptide synthesis and activating multiple immune signaling pathways (51).

## 4 Multi-strain probiotics

Multi-strain probiotics are gaining significant popularity in aquaculture because they have often yielded a greater overall impact on health than their single-strain counterparts. These multi-strain systems consist of multiple microbial species that will interact with the host organism as well as among each other to create enhanced synergistic effects. By interacting with multiple biological and immune pathways the various components of these blends have an extensive ability to enhance the health status of the aquatic organism. This means that these types of probiotics can improve an organism’s disease resistance as well as stimulate a more robust immune system (52).

Multi-strain probiotics are essentially blends containing at least two or more different species of microbial strains. Both individual species and several different species will make up the contents of the probiotic blends. The main intention when developing multi-strain probiotic blends is to choose the correct combinations of strains so that they exhibit positive attributes that benefit the host. These positive attribute could include increased digestion, an increase in resistance against disease-causing pathogens, or the stimulation of the host immune system. The most important characteristic of these probiotics is that they have the ability to remain compatible so that the strains do not inhibit the effectiveness of one another. Instead of stimulating the host’s immune response through only a few specific immune or biological pathways they are able to stimulate numerous at one time (53).

The effectiveness of these types of multi-strain probiotics are typically determined by synergistic, additive or antagonistic relationships between the different strains included. Synergistic interactions would be desirable when creating a multi-strain probiotic system as this type of relationship between strains would produce the highest beneficial effect in the host, such as resistance to pathogens. In an additive system, the addition of various probiotic strains would all provide separate benefits that simply sum up for an overall result. An antagonistic interaction between strains means that the strains are not conducive to growth within each other; they actually negatively impact the effectiveness of the particular probiotic system. It is crucial to have all interactions determined during the formulation process of multi-strain probiotic systems (54).

Multi-strain probiotic systems usually provide larger immunomodulatory effects at the molecular level than do single-strain probiotic products. Specifically it has been found that while these types of probiotics can cause an up-regulation of pro-inflammatory cytokines during early infection phases which enhances host detection and elimination of pathogenic bacteria they also create anti-inflammatory cytokine expression which prevents risks to over-inflation (55). As mentioned before multi-strain probiotics, such as the one utilizing Bacillus and Lactobacillus species in shrimp culture has demonstrated the capability to elicit strong improvements in host immune gene expression and also an enhancement in phenoloxidase activity. Additionally, the *Bacillus* and *Lactobacillus* strain combinations provided resistance to pathogens like *Vibrio*. For fin fish, using multi-strain probiotics results in the strengthening of innate and adaptive immunity which also translates to increased survival rates when under pathogenic pressure. It is important to consider these systems can aid to promote homeostasis within the immune system while preventing an overreaction that would result in the loss of healthy host cells (56). Although a single strain probiotic has predictable results and highly specific immune function, multi-strain probiotics act upon the host through multiple pathways in the body. For an effective product however, strains must be selected to work in concert, the correct dose of probiotic is administered, and the hosts environment and other contributing biological factors are taken into account. In the correct conditions these multi-strain probiotics hold vast promise for the health management sector of aquaculture.

While MSPs have some advantages over single-strain probiotics, they have potential shortcomings. Performance is based on the compatibility and symbiotic relationship between components because antagonistic effects of probiotics reduce performance of other beneficial probiotics in MSPs. The formulation complexity, environmental factors and storage stability of a multiple strain probiotics can result in varying performance (57).

## 5 Comparative analysis of single-strain vs. multi-strain probiotics

In order to investigate relative effectiveness between single and multiple strains on improving immunity in aquaculture, an evaluation comparing the two strategies is needed. Both single and multiple probiotic strains have shown effectiveness within aquaculture, but they differ significantly in terms of their fundamental biological processes, in the ways they interact with host microbiota, in their immune modulatory capabilities, and in how their performance responds to multiple environmental challenges. This section offers a cohesive and critical analysis of these differences, through the perspectives of theoretical, molecular, and applied considerations (57) (**Table 1**).

**Table 1:** Characteristics of single-strain and multi-strain probiotics in aquaculture species.

Animal model	Probiotic type	Example strains	Key immune effects	Microbiota impact	Disease resistance outcomE	References
Carp (*Cyprinus carpio*)	Single strain	*Bacillus subtilis*, *Lactobacillus plantarum*	Increased lysozyme, respiratory burst, moderate IL-1β and TNF-α expression	Limited, strain-specific modulation	Resistance to *Aeromonas hydrophila*	(58)
	Multi-strain	*Bacillus* + *Lactobacillus* spp.	Enhanced phagocytosis, cytokine upregulation, improved complement activity	Increased microbial diversity	Broad-spectrum pathogen resistance	(59)
Tilapia (*Oreochromis niloticus*)	Single-strain	*Bacillus coagulans*, *Bacillus subtilis*	Improved lysozyme, complement activity, moderate cytokine expression	Moderate microbiota modulation	Protection against *Streptococcus agalactiae*	(60)
	Multi-strain	*Bacillus* + *Lactobacillus* + *Enterococcus*	Strong IL-1β, IL-6, TNF-α expression; balanced IL-10 response	Significant restructuring of gut microbiota	Higher survival under infection stress	(61)
Pangasius (*Pangasianodon hypophthalmus*)	Single-strain	*Bacillus subtilis*	Improved antioxidant enzymes (SOD, CAT), moderate immune stimulation	Limited	Moderate disease resistance	(62)
	Multi-strain	Mixed *Bacillus* + LAB	Enhanced immune gene expression, stress tolerance	Improved gut stability	Increased survival under stress	(63)
Shrimp (*Litopenaeus vannamei*)	Single-strain	*Bacillus subtilis*, *Lactobacillus* spp.	Increased phenoloxidase, hemocyte count, moderate AMP expression	Limited microbial restructuring	Moderate resistance to *Vibrio* spp.	(64)
	Multi-strain	*Bacillus* + *Lactobacillus* + *Pseudomonas*	Strong activation of innate immunity, increased AMPs, phenoloxidase, cytokines	High microbial diversity and stability	High resistance to *Vibrio* infections	(65)
Crabs (*Scylla* spp.*)*	Single-strain	*Bacillus* spp.	Improved hemocyte activity and enzyme levels	Minimal	Moderate survival improvement	(66)
	Multi-strain	Mixed bacterial consortia	Enhanced immune enzyme activity, stress tolerance	Improved microbiota balance	Better survival under infection	(67)
Biofloc Systems (BFT)	Single-strain	*Bacillus subtilis*	Targeted immune enhancement (lysozyme, ROS)	Limited integration with floc microbiota	Moderate improvement	(68)
	Multi-strain	Mixed probiotic consortia	Enhanced cytokine expression, antioxidant activity, immune balance	Strong interaction with biofloc microbiome	High survival and system stability	(69)

Single-strain probiotics (SSPs) and multi-strain probiotics (MSPs) mediate immune modulation and pathogen control through distinct mechanisms of action (70). SSPs consist of a single well-characterized microbial strain that modulates the host immune response and inhibits pathogen colonization through specific, strain-dependent mechanisms, including competitive exclusion, antimicrobial metabolite production, and immune stimulation. In contrast, MSPs combine multiple functionally complementary probiotic strains to enhance microbial diversity, promote gut microbiota stability, strengthen competitive exclusion of pathogens, and coordinate multiple immune signaling pathways (71). Consequently, MSPs may provide greater resilience and broader protection against diverse environmental and pathogenic challenges. However, their effectiveness depends on the compatibility of constituent strains, appropriate environmental conditions, and optimized formulation to ensure synergistic interactions and consistent performance.

The comparative data available across fish and crustacean species suggest that the difference between SSPs and MSPs goes far beyond differences in the intensity of the immune response and reflect fundamental differences in host–microbe interaction mechanisms. SSPs typically act by means of targeted strain-specific mechanisms such as the production of antimicrobial compounds, exclusion of pathogens from the host site, the reinforcement of mucosal barrier function, the activation of innate immunity such as lysozyme production, respiratory burst activity, complement activation, and antioxidant defence (58, 60, 62, 64, 66, 68). Such direct mechanisms are ideal when a specific pathogen or physiological function needs to be tackled; however their spectrum of action is usually limited since they often depends on the metabolic capacity of a single organism. MSPs exert their function through a consortium of complementary and cooperative strains which provide synergistic effects at both ecosystem and host level.

A consortium can combine specific strain functionalities (nutrient utilization, diverse antimicrobial metabolite production, immune signalling modulation and stabilization of microbial populations) in a co-operative fashion to the point that they promote much more complex functions such as a wider range of antimicrobial compounds, increased microbial diversity, a more efficient barrier to colonization, and a much wider range of immune stimulatory responses (Cytokine production, antioxidant capacity, complement) compared with SSPs, providing thereby a greater level of protection against multiple opportunistic pathogens and general environmental stressors (59, 61, 63, 65, 67, 69). SSP and MSP difference can be clearly appreciated in the context of a biofloc system where the probiotic effectiveness also depend on the interaction with the resident biofloc microbiome. SSPs generally have an effect on a subset of immune parameters without largely disturbing the overall microbial communities of the biofloc (68), while MSPs tend to integrate more successfully within the mixed communities of a biofloc, contributing to functional redundancy, more efficient nutrient cycling, microbial stabilization and coordinated immune stimulation (69).

### 5.1 Conceptual perspective: targeted strategy vs. ecosystem engineering

Probiotics with a single-strain formulation fall within the category of a highly targeted approach (72). This strategy is based on selecting a single microorganism with well-understood functional properties, including enzyme production, immune stimulation, and antimicrobial activity (73). The functional profile and consequently, the resultant health effect of a single-strain probiotic is usually predictable and reproducible since biological pathways of a single-strain are well understood. For example, a certain strain may be incorporated for the purpose of eliminating a particular pathogen or enhancing a specific immune component, lysozyme (74).

Probiotics with a mixture of multiple strains are developed on the principle of a comprehensive ecosystem engineering. Instead of one specific function, the mixture consists of several species in a community that interact together (75). These mixtures try to mimic natural communities of microorganisms in order to perform a variety of biological functions including nutritional processing, pathogen exclusion and modulation of immunity (76). However, the overall functional effects of these species depends on the interaction between themselves and whether they are synergistic or competitive. Hence, such multi-strain consortia possess improved functional versatility and stability against variable environmental conditions.

### 5.2 Comparison of functional properties

In light of the above discussion, fundamental functional characteristics of single strain versus multi-strain probiotics are compared based on key parameters (**Table 2**) (15).

**Table 2:** Comparative characteristics of single-strain versus multi-strain probiotics in aquaculture.

Parameter	Single-strain probiotics	Multi-strain probiotics
Core Concept	Targeted, strain-specific action	Ecosystem-based, multi-functional approach
Mechanism of Action	Defined and pathway-specific	Synergistic and complementary mechanisms
Immune Response	Moderate, focused activation	Stronger, multi-pathway immune stimulation
Gut Microbiota Impact	Limited to specific niches	Broad restructuring and increased diversity
Stability and Predictability	High consistency and reproducibility	Variable, depends on strain compatibility
Disease Resistance	Effective against specific pathogens	Broad-spectrum protection against multiple pathogens
Immune Homeostasis	May induce localized responses	Better regulation of pro- and anti-inflammatory balance
Environmental Adaptability	Performs best in controlled systems	More resilient in fluctuating environments
Application Complexity	Simple formulation and standardization	Requires careful design and optimization

The single-strain probiotics (SSPs) and multi-strain probiotics (MSPs) have different beneficial impacts predominantly because of different modes of action (71). The SSPs have direct and predictable outcomes that can be attributed to strain-specific mechanisms of action in order to be appropriate for closed and controlled farming practices (77). Conversely, MSPs integrate different organisms that complement each other to act as synergists to provide improved biodiversity in gut microbiome, superior competitive exclusion of pathogens and a widespread increase in immune responses. These synergistic mechanisms provide for improved immune regulation, stronger immunity and more favourable environmental adaptations (15).

**Table 2** indicates that based on their interactions with host microbiota, SSPs and MSPs differ mainly in functional mechanisms. SSPs exert their functions through the well-established activity of a unique species that target specific effects by, for example, outcompeting pathogens, antagonizing them through microbial metabolites, bolstering intestinal barrier functions and triggering specific innate immune responses (58, 60, 62). This targeted approach ensures highly predictable performance and reproducibility but potentially compromises a broad range of immuno-stimulation and adaptability in various culture conditions (68). MSPs, on the other hand, introduce several strains that perform complementary functions and work in unison to alter both host immune responses and the composition of gut microbiota (59, 61, 63). These collective effects enhance gut microbiota diversity, improve resistance to colonization and modulate multiple innate and adaptive immunity mechanisms at the same time, providing balanced pro-inflammatory and anti-inflammatory reactions in order to counter the effects of both environmental stresses and polymicrobial infections (65, 67, 69). The success of MSP depends on several conditions including compatible strains and synergism among introduced microbiota, as antagonistic effect might be found among inappropriate strains, leading to lower probiotic effects (71). Thus, a proper choice between SSP and MSP should be made based on culture applications, environment and pathogen profiles, not on the basis that one type of probiotics is generally superior to the other.

However, effectiveness of MSPs could be limited in the event of inappropriate composition of strains, antagonism among microbes or in the face of unfavourable conditions. In any case, decisions should be made considering the species, cultivation system, required effects on the immunity and production and favourable environmental settings (65).

## 6 Synergistic integration of Biofloc technology and probiotic strategies

One of the most promising innovations for sustainable aquaculture is the integration of probiotics with Biofloc Technology (BFT) (78). Biofloc technology provides a system that inherently promotes high densities of microbial communities capable of converting waste nutrients into food and maintaining water quality (79). When probiotics are introduced to such systems, they must interact with the existing microbial communities to effect the host’s health and the stability of the entire ecosystem.

Probiotics introduced as single strains to BFT systems have been shown to have limited specific and directed responses (80). It can be argued that because of the diverse and complex nature of BFT microbial communities that the effects of single strains are diluted. Multi-strain probiotics have been shown to be most successful with BFT systems because they are often more readily accepted by the complex microbial communities present in these systems. Because multi-strain probiotics have a cooperative effect, they readily establish and support a more readily integrated, organic matter degradation, nutrient cycling, and disease-antagonism BFT environment (81). Using BFT systems with multi-strain probiotics is recognized as stimulating strong host innate immunity. Aquatic organisms grown in BFT systems with multi-strain probiotics showed increased lysozyme activity, antioxidant ability and immune gene expression (19). Probiotic administration benefits by naturally facilitating nitrification, utilizing organic matter for host benefits and for lowering levels of toxic ammonia and nitrite. Combined host immune stimulating and water quality benefits make BFT systems and multi-strain probiotics beneficial for aquaculture (82).

This synergy between the addition of multi-strain probiotic consortia (MSPs) and biofloc technology (BFT) is due to the symbiotic interaction of exogenous probiotics and indigenous microorganisms in the biofloc. Various probiotic strains have the ability to perform distinct roles in breakdown of organic matter, nitification, inhibiting pathogens and also synthesizing of valuable substances while the biofloc provides a favorable niche to adhere and colonize the introduced bacteria. It leads to recycle of nutrient, control of pathogenic microorganisms by competing with native bacteria for habitat and food (competitive exclusion) and production of useful metabolites, in which cultured animals gain a stable environment with a more balanced microbiota structure and increased tolerance against stress and pathogens and higher efficiency. Nevertheless, these benefits can be modified depending on the interaction between probiotic strains and native microorganisms in the bioflocs, nature of carbon source, and biofloc system (69).

## 7 Future perspectives

Probiotics applied in aquaculture has been developing from simply applying methods, to highly tailored and directed approaches that may seem beyond the traditional principles of probiotic application. One of the most significant fields of future research, involves the ‘design’ of microbial communities having a particular set of specific functions that synergize with maximum advantage to the host. The use of computational biology and AI to achieve such complex design will facilitate selections of strains with combined specific functions, and predictions on the microbial interactions (83). The combined application of pre- and probiotics are demonstrated to lead to better immune response, as probiotics require nutrition to multiply and grow. Postbiotics (bioactive metabolites produced by the probiotics) is developed as a secure and stable approach for immune modulation (84). In addition, host-specific probiotics are also on the way aiming at promoting health of a specific host’s immune system. ‘Omics’ such as genomics, transcriptomics and metabolomics become useful tools in studying the interactions between host and its microbes for optimal health and production (53).

## 8 Conclusion

Probiotics have emerged as a safe, stable, and efficient means to enhance immunity and disease resistance in aquaculture. The primary differences between single-strain and multi-strain probiotics lie in their mechanisms of action, immunomodulatory effects, and practical applications in aquaculture. Probiotics based on one single strain may result in very precise outcomes, in controlled and defined conditions in which standardization and precise delivery is achieved. Probiotics consisting of many different strains can yield more diverse and enhanced effects. Multi-strain probiotic compositions often result in increased efficiency through synergistic outcomes on gut microflora and numerous parts of the immune system (53). It is agreed that a positive effect of multi-strain preparations on immunity and gut microflora compared to single-strain preparation is evident in the scientific literature, however it is still impossible to confirm it without reserve because it all depends on the ability of strains to cope, the species being treated and the environment. In a great number of cases, single-strain probiotic is as effective, if not better, than its multi-strain counterpart in the specific target pathogen and controlled operating procedure environment. Another way to further enhance probiotic benefits in aquaculture practices is to combine them with other technologies such as BFT where an overall immune system strengthening can be combined with broader microbiological influence on water quality.
